# *N*′-Phenyl-*N*′-[3-(2,4,5-triphenyl-2,5-di­hydro-1*H*-pyrazol-3-yl)quinoxalin-2-yl]benzohydrazide

**DOI:** 10.1107/S1600536813020035

**Published:** 2013-07-27

**Authors:** Youssef Ramli, Khalid Karrouchi, El Mokhtar Essassi, Lahcen El Ammari

**Affiliations:** a Laboratoire National de Contrôle des Médicaments, D M P, Ministère de la Santé, Madinat Al Irnane, BP 6206, Rabat, Morocco; b Laboratoire de Chimie Thérapeutique, Faculté de Médecine et de Pharmacie de Rabat-Souissi, Université Mohamed V, BP 6203, Rabat, Morocco; c Laboratoire de Chimie Organique Hétérocyclique URAC21, Pôle de Compétences Pharmacochimie, Université Mohammed V-Agdal, Avenue Ibn Battouta, BP 1014, Rabat, Morocco; d Institute of Nanomaterials and Nanotechnology, MASCIR, Rabat, Morocco; e Laboratoire de Chimie du Solide Appliquée, Université Mohammed V-Agdal, Faculté des Sciences, Avenue Ibn Battouta, BP 1014, Rabat, Morocco

## Abstract

The mol­ecule of the title compound, C_42_H_32_N_6_O, is built up from one pyrazole ring linked to three phenyl rings and to an approximately planar [maximum deviation = 0.0455 (15) Å] quinoxaline system connected to a phenyl­benzohydrazide group. The pyrazole ring assumes an envelope conformation, the C atom attached to the quinoxalin-3-yl ring system being the flap atom. The dihedral angle between the two phenyl rings of the phenyl­benzohydrazide group is of 58.27 (9)°. The mean plane through the pyrazole ring is nearly perpendicular to the quinoxaline ring system and to the phenyl ring attached to the opposite side, forming dihedral angles of 82.58 (7) and 87.29 (9)°, respectively. An intra­molecular C—H⋯O hydrogen bond is present. In the crystal, mol­ecules are linked by pairs of N—H⋯N hydrogen bonds, forming inversion dimers, which are further connected by C—H⋯N hydrogen bonds into chains parallel to the *b* axis.

## Related literature

For the biological activity of quinoxaline derivatives, see: El-Sabbagh *et al.* (2009 ▶); Bemis & Duffy (2005 ▶); Corona *et al.* (2008 ▶); Ghadage & Shirote (2011 ▶); Yang *et al.* (2012 ▶).
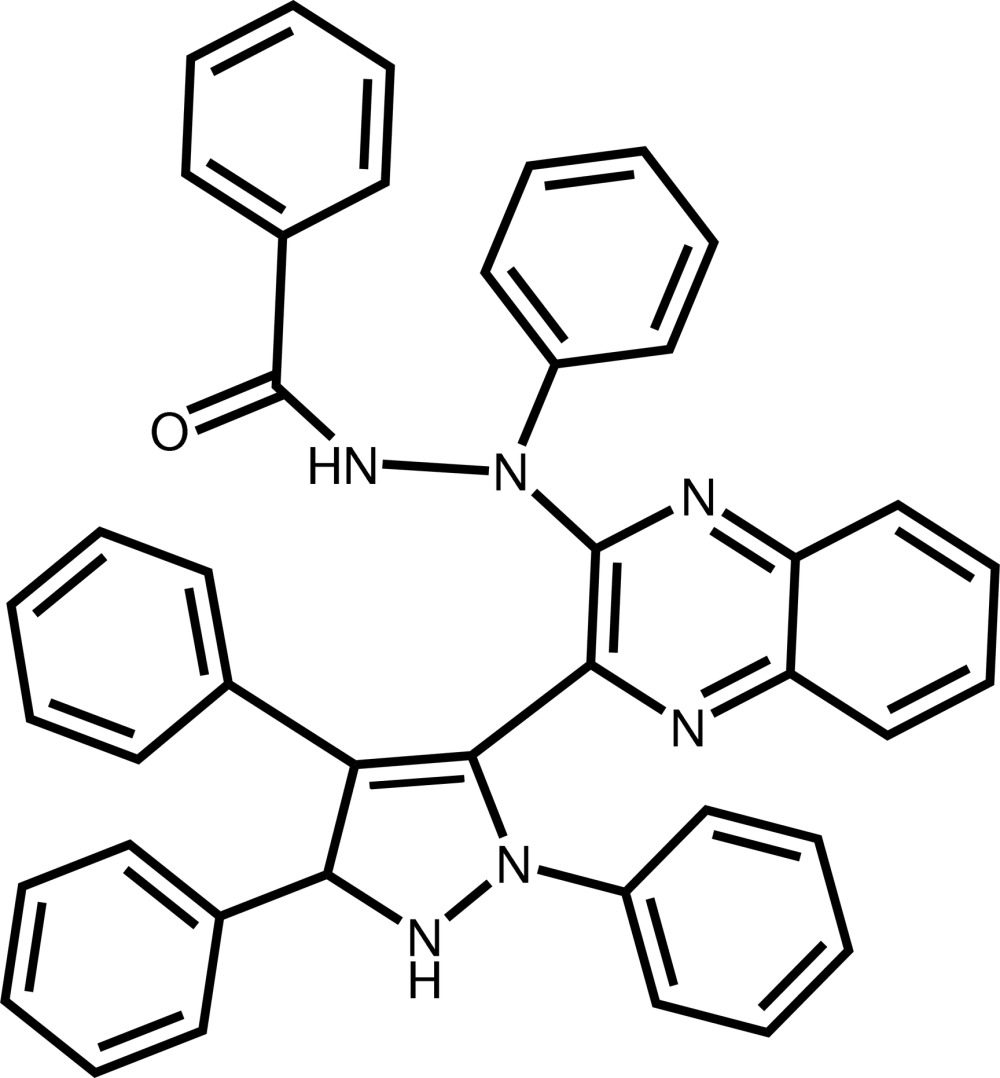


## Experimental

### 

#### Crystal data

C_42_H_32_N_6_O
*M* *_r_* = 636.74
Monoclinic, 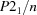

*a* = 12.0127 (3) Å
*b* = 19.4679 (5) Å
*c* = 15.2083 (4) Å
β = 106.045 (1)°
*V* = 3418.09 (15) Å^3^
*Z* = 4
Mo *K*α radiation
μ = 0.08 mm^−1^
*T* = 296 K
0.41 × 0.32 × 0.21 mm

#### Data collection

Bruker X8 APEXII area-detector diffractometer
39727 measured reflections
8306 independent reflections
4919 reflections with *I* > 2σ(*I*)
*R*_int_ = 0.050

#### Refinement

*R*[*F* ^2^ > 2σ(*F* ^2^)] = 0.047
*wR*(*F* ^2^) = 0.117
*S* = 1.00
8306 reflections
443 parameters
H-atom parameters constrained
Δρ_max_ = 0.20 e Å^−3^
Δρ_min_ = −0.19 e Å^−3^

### 

Data collection: *APEX2* (Bruker, 2009 ▶); cell refinement: *SAINT* (Bruker, 2009 ▶); data reduction: *SAINT*; program(s) used to solve structure: *SHELXS97* (Sheldrick, 2008 ▶); program(s) used to refine structure: *SHELXL97* (Sheldrick, 2008 ▶); molecular graphics: *ORTEP-3 for Windows* (Farrugia, 2012 ▶); software used to prepare material for publication: *PLATON* (Spek, 2009 ▶) and *publCIF* (Westrip, 2010 ▶).

## Supplementary Material

Crystal structure: contains datablock(s) I. DOI: 10.1107/S1600536813020035/rz5080sup1.cif

Structure factors: contains datablock(s) I. DOI: 10.1107/S1600536813020035/rz5080Isup2.hkl

Supplementary material file. DOI: 10.1107/S1600536813020035/rz5080Isup3.cml

Additional supplementary materials:  crystallographic information; 3D view; checkCIF report

## Figures and Tables

**Table 1:** Hydrogen-bond geometry (Å, °)

*D*—H⋯*A*	*D*—H	H⋯*A*	*D*⋯*A*	*D*—H⋯*A*
C2—H2⋯O3	0.98	2.28	3.2425 (19)	169
N6—H6*N*⋯N4^i^	0.89	2.30	3.1754 (18)	172
C16—H16⋯N2^ii^	0.93	2.56	3.4799 (19)	172

Symmetry codes: (i) 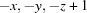; (ii) 
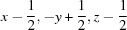
.

**Fig. 1.**
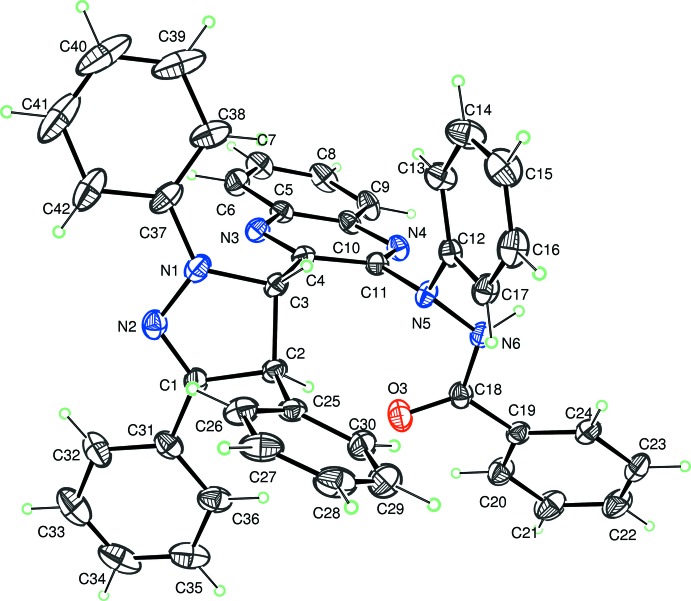
The molecular structure of the title compound with displacement ellipsoids drawn at the 50% probability level. H atoms are represented as small circles of arbitrary radii.

## supplementary crystallographic information

### Comment

Quinoxalinone and its derivatives are used in organic synthesis for building natural and designed synthetic compounds and have been frequently utilized as suitable skeletons for the design of biologically active compounds. For instance, they are known for their potent activity as anti-inflammatory agents (El-Sabbagh *et al.*, 2009), inhibitors of the kinase protein (Bemis *et al.*, 2005), anti-cancer agents (Corona *et al.*, 2008), anti-microbial agents (Ghadage & Shirote, 2011) and are particularly effective in the treatment of diabetes and its complications (Yang *et al.*, 2012). Our research group targeted at the development of novel quinoxalinone derivatives such as the title compound that may prove to be better agents in terms of efficacy and safety.

The molecule of the title compound displays a five membered pyrazol-5-yl ring (N1/N2/C1–C3) connected to three phenyl rings and to a quinoxalin-3-yl ring system attached to a phenylbenzohydrazide group (Fig. 1). The pyrazole ring shows an envelope conformation as indicated by the total puckering amplitude QT = 0.2552 (16) Å and spherical polar angle φ2 = 135.1 (4)°. The C3 flap atom is displaced by 0.4035 (14) Å from the mean plane through the other four atoms. The fused quinoxalin-3-yl ring system (N3/N4/C4–C11) is approximately planar with a maximum deviation from the mean plane of 0.0455 (15) Å at C11. The dihedral angle between the two phenyl rings (C12–C17 and C14–C24) of the phenylbenzohydrazide moiety is 58.27 (9)°. The mean plane through the five-membered pyrazole ring is nearly perpendicular to the quinoxaline ring system and to the C25–C30 phenyl ring as indicated by the dihedral angles of 82.58 (7)° and 87.29 (9)°, respectively. It makes also dihedral angles of 14.38 (9)° and 17.82 (9)° with the attached C31–C36 and C37–C42 phenyl rings, respectively. An intramolecular C—H···O hydrogen bond is observed (Table 1). In the crystal, centrosymmetrically related molecules are linked by pairs of N—H···N hydrogen bonds (Table 1) into dimers. The dimers are further linked into chains parallel to the *b* axis by C—H···N hydrogen bonds.

### Experimental

A mixture of α-chlorobenzylidene phenylhydrazine (8.1 mmol) and triethylamine (8.1 mmol) in THF (40 mL) was added at room temperature to a solution of 3-styryl-quinoxalin-2-one (6.5 mmol) in THF (20 mL). The reaction mixture was heated under reflux for 48 h. The inorganic salts formed were filtered off. The filtrate was evaporated under reduced pressure and the crude product obtained was recrystallized from ethanol to afford crystals of the title compound.

### Refinement

All H atoms could be located in a difference Fourier map and were treated as riding with C—H = 0.93 Å, N—H = 0.89 Å and with *U*_iso_(H) = 1.2 *U*_eq_(C, N). Six reflections affected by the beam stop were omitted because the difference between their calculated and observed intensities was very large.

### Crystal data

**Table:**

C_42_H_32_N_6_O	*F*(000) = 1336
*M**_r_* = 636.74	*D*_x_ = 1.237 Mg m^−^^3^
Monoclinic, *P*2_1_/*n*	Mo *K*α radiation, λ = 0.71073 Å
Hall symbol: -P 2yn	Cell parameters from 8306 reflections
*a* = 12.0127 (3) Å	θ = 2.7–28.2°
*b* = 19.4679 (5) Å	µ = 0.08 mm^−^^1^
*c* = 15.2083 (4) Å	*T* = 296 K
β = 106.045 (1)°	Block, colourless
*V* = 3418.09 (15) Å^3^	0.41 × 0.32 × 0.21 mm
*Z* = 4	

### Data collection

**Table:**

Bruker X8 APEXII area-detector diffractometer	4919 reflections with *I* > 2σ(*I*)
Radiation source: fine-focus sealed tube	*R*_int_ = 0.050
Graphite monochromator	θ_max_ = 28.2°, θ_min_ = 2.7°
φ and ω scans	*h* = −15→15
39727 measured reflections	*k* = −25→25
8306 independent reflections	*l* = −20→17

### Refinement

**Table:**

Refinement on *F*^2^	Secondary atom site location: difference Fourier map
Least-squares matrix: full	Hydrogen site location: difference Fourier map
*R*[*F*^2^ > 2σ(*F*^2^)] = 0.047	H-atom parameters constrained
*wR*(*F*^2^) = 0.117	*w* = 1/[σ^2^(*F*_o_^2^) + (0.0478*P*)^2^ + 0.4886*P*] where *P* = (*F*_o_^2^ + 2*F*_c_^2^)/3
*S* = 1.00	(Δ/σ)_max_ < 0.001
8306 reflections	Δρ_max_ = 0.20 e Å^−^^3^
443 parameters	Δρ_min_ = −0.19 e Å^−^^3^
0 restraints	Extinction correction: *SHELXL97* (Sheldrick, 2008), Fc^*^=kFc[1+0.001xFc^2^λ^3^/sin(2θ)]^-1/4^
Primary atom site location: structure-invariant direct methods	Extinction coefficient: 0.0019 (4)

### Special details

**Table:**

Geometry. All s.u.'s (except the s.u. in the dihedral angle between two l.s. planes) are estimated using the full covariance matrix. The cell s.u.'s are taken into account individually in the estimation of s.u.'s in distances, angles and torsion angles; correlations between s.u.'s in cell parameters are only used when they are defined by crystal symmetry. An approximate (isotropic) treatment of cell s.u.'s is used for estimating s.u.'s involving l.s. planes.
Refinement. Refinement of *F*^2^ against all reflections. The weighted *R*-factor *wR* and goodness of fit *S* are based on *F*^2^, conventional *R*-factors *R* are based on *F*, with *F* set to zero for negative *F*^2^. The threshold expression of *F*^2^ > σ(*F*^2^) is used only for calculating *R*-factors(gt) *etc*. and is not relevant to the choice of reflections for refinement. *R*-factors based on *F*^2^ are statistically about twice as large as those based on *F*, and *R*- factors based on all data will be even larger.

### Fractional atomic coordinates and isotropic or equivalent isotropic displacement parameters (Å^2^)

**Table:**

	*x*	*y*	*z*	*U*_iso_*/*U*_eq_	
C1	0.13275 (13)	0.33151 (8)	0.47673 (10)	0.0316 (3)	
C2	0.06730 (13)	0.27133 (8)	0.42495 (9)	0.0297 (3)	
H2	0.1159	0.2304	0.4300	0.036*	
C3	−0.02281 (13)	0.26028 (7)	0.47999 (9)	0.0274 (3)	
H3	−0.0999	0.2478	0.4403	0.033*	
C4	0.02100 (12)	0.20572 (8)	0.55295 (9)	0.0267 (3)	
C5	0.10980 (12)	0.17641 (8)	0.70169 (9)	0.0299 (3)	
C6	0.15065 (14)	0.19551 (9)	0.79455 (10)	0.0412 (4)	
H6	0.1456	0.2410	0.8118	0.049*	
C7	0.19740 (15)	0.14740 (10)	0.85869 (11)	0.0467 (5)	
H7	0.2221	0.1599	0.9201	0.056*	
C8	0.20864 (15)	0.07971 (10)	0.83350 (11)	0.0482 (5)	
H8	0.2426	0.0476	0.8782	0.058*	
C9	0.17070 (14)	0.05958 (9)	0.74423 (11)	0.0430 (4)	
H9	0.1795	0.0142	0.7281	0.052*	
C10	0.11820 (13)	0.10795 (8)	0.67679 (10)	0.0310 (4)	
C11	0.02421 (12)	0.13436 (8)	0.52876 (9)	0.0278 (3)	
C12	−0.14430 (13)	0.11813 (7)	0.39499 (10)	0.0295 (3)	
C13	−0.22002 (14)	0.11864 (9)	0.44837 (11)	0.0399 (4)	
H13	−0.1918	0.1180	0.5118	0.048*	
C14	−0.33859 (15)	0.12013 (10)	0.40724 (13)	0.0529 (5)	
H14	−0.3896	0.1213	0.4434	0.064*	
C15	−0.38114 (16)	0.11984 (10)	0.31384 (13)	0.0527 (5)	
H15	−0.4607	0.1196	0.2867	0.063*	
C16	−0.30551 (16)	0.11984 (9)	0.26043 (11)	0.0463 (5)	
H16	−0.3343	0.1200	0.1970	0.056*	
C17	−0.18744 (15)	0.11955 (8)	0.30003 (10)	0.0369 (4)	
H17	−0.1368	0.1203	0.2635	0.044*	
C18	0.14618 (14)	0.07099 (8)	0.40332 (10)	0.0336 (4)	
C19	0.20182 (14)	0.01078 (8)	0.37266 (10)	0.0325 (4)	
C20	0.32110 (15)	0.00497 (9)	0.40344 (12)	0.0454 (4)	
H20	0.3639	0.0382	0.4424	0.055*	
C21	0.37670 (17)	−0.04982 (11)	0.37664 (13)	0.0565 (5)	
H21	0.4569	−0.0536	0.3978	0.068*	
C22	0.31412 (18)	−0.09881 (11)	0.31888 (14)	0.0588 (5)	
H22	0.3518	−0.1360	0.3015	0.071*	
C23	0.19607 (18)	−0.09299 (10)	0.28676 (13)	0.0553 (5)	
H23	0.1539	−0.1260	0.2472	0.066*	
C24	0.13965 (15)	−0.03813 (9)	0.31305 (11)	0.0409 (4)	
H24	0.0597	−0.0341	0.2906	0.049*	
C25	0.01238 (13)	0.28786 (8)	0.32437 (10)	0.0320 (4)	
C26	−0.03921 (14)	0.35066 (9)	0.29610 (11)	0.0416 (4)	
H26	−0.0372	0.3850	0.3390	0.050*	
C27	−0.09401 (15)	0.36282 (11)	0.20433 (12)	0.0502 (5)	
H27	−0.1286	0.4052	0.1862	0.060*	
C28	−0.09737 (16)	0.31247 (12)	0.14017 (12)	0.0543 (5)	
H28	−0.1353	0.3204	0.0789	0.065*	
C29	−0.04462 (17)	0.25073 (11)	0.16706 (12)	0.0561 (5)	
H29	−0.0458	0.2168	0.1237	0.067*	
C30	0.01064 (15)	0.23837 (9)	0.25863 (11)	0.0437 (4)	
H30	0.0469	0.1964	0.2760	0.052*	
C31	0.24876 (14)	0.35178 (9)	0.47640 (11)	0.0392 (4)	
C32	0.30152 (18)	0.40906 (10)	0.52508 (15)	0.0610 (6)	
H32	0.2604	0.4363	0.5555	0.073*	
C33	0.4142 (2)	0.42592 (13)	0.52862 (17)	0.0765 (7)	
H33	0.4485	0.4643	0.5616	0.092*	
C34	0.47581 (19)	0.38669 (14)	0.48410 (16)	0.0713 (7)	
H34	0.5516	0.3986	0.4865	0.086*	
C35	0.42580 (17)	0.32967 (13)	0.43584 (13)	0.0617 (6)	
H35	0.4681	0.3028	0.4060	0.074*	
C36	0.31229 (15)	0.31201 (10)	0.43149 (11)	0.0485 (5)	
H36	0.2787	0.2735	0.3985	0.058*	
C37	−0.09873 (14)	0.35058 (9)	0.57148 (10)	0.0412 (4)	
C38	−0.17521 (16)	0.30522 (11)	0.59395 (11)	0.0528 (5)	
H38	−0.1776	0.2596	0.5754	0.063*	
C39	−0.24821 (19)	0.32803 (16)	0.64427 (14)	0.0816 (8)	
H39	−0.2991	0.2972	0.6596	0.098*	
C40	−0.2468 (2)	0.3944 (2)	0.67158 (17)	0.1044 (12)	
H40	−0.2959	0.4090	0.7056	0.125*	
C41	−0.1725 (2)	0.43964 (16)	0.64864 (17)	0.0931 (10)	
H41	−0.1718	0.4852	0.6671	0.112*	
C42	−0.09776 (17)	0.41894 (11)	0.59825 (13)	0.0621 (6)	
H42	−0.0480	0.4503	0.5827	0.075*	
N1	−0.02553 (12)	0.32888 (7)	0.51901 (8)	0.0360 (3)	
N2	0.07573 (12)	0.36378 (7)	0.52524 (8)	0.0365 (3)	
N3	0.06255 (10)	0.22567 (6)	0.63778 (8)	0.0305 (3)	
N4	0.07247 (10)	0.08697 (6)	0.58766 (8)	0.0322 (3)	
N5	−0.02142 (10)	0.11631 (6)	0.43538 (8)	0.0301 (3)	
N6	0.03408 (11)	0.06146 (6)	0.40476 (8)	0.0315 (3)	
H6N	0.0090	0.0199	0.4130	0.038*	
O3	0.19716 (10)	0.12528 (6)	0.42520 (9)	0.0483 (3)	

### Atomic displacement parameters (Å^2^)

**Table:**

	*U*^11^	*U*^22^	*U*^33^	*U*^12^	*U*^13^	*U*^23^
C1	0.0384 (9)	0.0283 (8)	0.0266 (7)	−0.0008 (7)	0.0065 (6)	0.0005 (6)
C2	0.0350 (8)	0.0305 (9)	0.0258 (7)	0.0031 (7)	0.0120 (6)	0.0010 (6)
C3	0.0316 (8)	0.0283 (8)	0.0227 (7)	0.0024 (6)	0.0080 (6)	−0.0022 (6)
C4	0.0265 (8)	0.0307 (8)	0.0243 (7)	0.0003 (6)	0.0092 (6)	0.0001 (6)
C5	0.0289 (8)	0.0351 (9)	0.0246 (7)	−0.0037 (7)	0.0056 (6)	0.0023 (6)
C6	0.0477 (10)	0.0451 (10)	0.0278 (8)	−0.0042 (8)	0.0055 (7)	−0.0024 (7)
C7	0.0504 (11)	0.0584 (13)	0.0251 (8)	−0.0131 (9)	0.0003 (7)	0.0044 (8)
C8	0.0462 (11)	0.0504 (12)	0.0384 (9)	−0.0104 (9)	−0.0042 (8)	0.0183 (8)
C9	0.0456 (10)	0.0337 (10)	0.0415 (9)	−0.0068 (8)	−0.0019 (8)	0.0072 (7)
C10	0.0283 (8)	0.0335 (9)	0.0280 (8)	−0.0065 (7)	0.0025 (6)	0.0024 (6)
C11	0.0257 (8)	0.0323 (9)	0.0247 (7)	−0.0026 (6)	0.0056 (6)	−0.0019 (6)
C12	0.0321 (8)	0.0243 (8)	0.0289 (7)	−0.0007 (6)	0.0031 (6)	0.0003 (6)
C13	0.0368 (10)	0.0503 (11)	0.0298 (8)	−0.0034 (8)	0.0046 (7)	0.0070 (7)
C14	0.0336 (10)	0.0737 (14)	0.0501 (11)	−0.0021 (9)	0.0093 (8)	0.0127 (10)
C15	0.0355 (10)	0.0604 (13)	0.0514 (11)	0.0016 (9)	−0.0057 (8)	0.0078 (9)
C16	0.0513 (11)	0.0452 (11)	0.0317 (9)	0.0052 (9)	−0.0066 (8)	0.0000 (8)
C17	0.0449 (10)	0.0352 (9)	0.0271 (8)	0.0021 (7)	0.0044 (7)	−0.0008 (7)
C18	0.0358 (9)	0.0328 (9)	0.0300 (8)	−0.0011 (7)	0.0055 (6)	−0.0008 (7)
C19	0.0385 (9)	0.0339 (9)	0.0263 (7)	0.0015 (7)	0.0109 (6)	0.0008 (6)
C20	0.0406 (10)	0.0531 (12)	0.0404 (9)	0.0029 (9)	0.0077 (8)	−0.0092 (8)
C21	0.0453 (11)	0.0694 (14)	0.0545 (11)	0.0153 (10)	0.0134 (9)	−0.0075 (10)
C22	0.0640 (14)	0.0554 (13)	0.0649 (13)	0.0130 (11)	0.0310 (11)	−0.0118 (10)
C23	0.0609 (13)	0.0563 (13)	0.0550 (11)	−0.0055 (10)	0.0268 (10)	−0.0256 (10)
C24	0.0436 (10)	0.0463 (11)	0.0359 (9)	−0.0039 (8)	0.0162 (7)	−0.0084 (8)
C25	0.0326 (8)	0.0401 (10)	0.0255 (7)	−0.0034 (7)	0.0118 (6)	0.0011 (7)
C26	0.0435 (10)	0.0490 (11)	0.0355 (9)	0.0079 (8)	0.0165 (7)	0.0055 (8)
C27	0.0403 (10)	0.0705 (13)	0.0422 (10)	0.0101 (9)	0.0155 (8)	0.0204 (9)
C28	0.0460 (11)	0.0864 (16)	0.0278 (9)	−0.0107 (11)	0.0058 (8)	0.0084 (10)
C29	0.0724 (14)	0.0671 (14)	0.0277 (9)	−0.0174 (11)	0.0118 (9)	−0.0082 (9)
C30	0.0582 (11)	0.0427 (10)	0.0316 (9)	−0.0071 (9)	0.0149 (8)	−0.0038 (7)
C31	0.0411 (10)	0.0404 (10)	0.0326 (8)	−0.0055 (8)	0.0045 (7)	0.0098 (7)
C32	0.0616 (13)	0.0434 (12)	0.0770 (14)	−0.0163 (10)	0.0174 (11)	−0.0011 (10)
C33	0.0644 (16)	0.0673 (16)	0.0924 (18)	−0.0295 (13)	0.0124 (13)	0.0041 (13)
C34	0.0465 (13)	0.0953 (19)	0.0690 (14)	−0.0217 (13)	0.0107 (11)	0.0265 (13)
C35	0.0463 (12)	0.0937 (17)	0.0469 (11)	−0.0061 (12)	0.0158 (9)	0.0149 (11)
C36	0.0431 (10)	0.0685 (13)	0.0335 (9)	−0.0076 (9)	0.0099 (8)	0.0054 (9)
C37	0.0419 (10)	0.0559 (11)	0.0213 (7)	0.0202 (9)	0.0015 (7)	−0.0044 (7)
C38	0.0468 (11)	0.0794 (15)	0.0353 (9)	0.0193 (10)	0.0163 (8)	0.0075 (9)
C39	0.0543 (13)	0.152 (3)	0.0448 (12)	0.0297 (15)	0.0233 (10)	−0.0010 (14)
C40	0.0635 (17)	0.186 (4)	0.0622 (16)	0.045 (2)	0.0141 (13)	−0.0477 (19)
C41	0.0745 (18)	0.121 (2)	0.0693 (16)	0.0436 (17)	−0.0047 (13)	−0.0553 (16)
C42	0.0590 (13)	0.0691 (14)	0.0478 (11)	0.0231 (11)	−0.0027 (9)	−0.0262 (10)
N1	0.0459 (8)	0.0333 (8)	0.0305 (7)	0.0053 (6)	0.0134 (6)	−0.0026 (6)
N2	0.0450 (8)	0.0322 (8)	0.0295 (7)	−0.0006 (6)	0.0057 (6)	−0.0005 (6)
N3	0.0335 (7)	0.0341 (8)	0.0233 (6)	0.0004 (6)	0.0065 (5)	−0.0008 (5)
N4	0.0326 (7)	0.0299 (7)	0.0308 (7)	−0.0031 (6)	0.0035 (5)	0.0007 (6)
N5	0.0322 (7)	0.0304 (7)	0.0256 (6)	0.0025 (6)	0.0044 (5)	−0.0066 (5)
N6	0.0371 (7)	0.0268 (7)	0.0308 (7)	−0.0005 (6)	0.0097 (5)	−0.0047 (5)
O3	0.0401 (7)	0.0346 (7)	0.0697 (8)	−0.0045 (6)	0.0146 (6)	−0.0104 (6)

### Geometric parameters (Å, º)

**Table:**

C1—N2	1.2990 (19)	C21—C22	1.372 (3)
C1—C31	1.450 (2)	C21—H21	0.9300
C1—C2	1.506 (2)	C22—C23	1.371 (3)
C2—C25	1.5235 (19)	C22—H22	0.9300
C2—C3	1.555 (2)	C23—C24	1.382 (2)
C2—H2	0.9784	C23—H23	0.9300
C3—N1	1.4655 (19)	C24—H24	0.9300
C3—C4	1.5211 (19)	C25—C30	1.384 (2)
C3—H3	0.9858	C25—C26	1.384 (2)
C4—N3	1.3071 (17)	C26—C27	1.389 (2)
C4—C11	1.440 (2)	C26—H26	0.9300
C5—N3	1.3717 (18)	C27—C28	1.376 (3)
C5—C10	1.397 (2)	C27—H27	0.9300
C5—C6	1.411 (2)	C28—C29	1.368 (3)
C6—C7	1.357 (2)	C28—H28	0.9300
C6—H6	0.9300	C29—C30	1.388 (2)
C7—C8	1.389 (3)	C29—H29	0.9300
C7—H7	0.9300	C30—H30	0.9300
C8—C9	1.365 (2)	C31—C32	1.391 (2)
C8—H8	0.9300	C31—C36	1.392 (2)
C9—C10	1.407 (2)	C32—C33	1.379 (3)
C9—H9	0.9300	C32—H32	0.9300
C10—N4	1.3764 (18)	C33—C34	1.366 (3)
C11—N4	1.3049 (18)	C33—H33	0.9300
C11—N5	1.4178 (17)	C34—C35	1.374 (3)
C12—C13	1.377 (2)	C34—H34	0.9300
C12—C17	1.393 (2)	C35—C36	1.390 (3)
C12—N5	1.4340 (19)	C35—H35	0.9300
C13—C14	1.389 (2)	C36—H36	0.9300
C13—H13	0.9300	C37—C38	1.384 (3)
C14—C15	1.370 (2)	C37—C42	1.391 (3)
C14—H14	0.9300	C37—N1	1.406 (2)
C15—C16	1.376 (3)	C38—C39	1.387 (3)
C15—H15	0.9300	C38—H38	0.9300
C16—C17	1.379 (2)	C39—C40	1.357 (4)
C16—H16	0.9300	C39—H39	0.9300
C17—H17	0.9300	C40—C41	1.366 (4)
C18—O3	1.2207 (18)	C40—H40	0.9300
C18—N6	1.365 (2)	C41—C42	1.391 (3)
C18—C19	1.487 (2)	C41—H41	0.9300
C19—C24	1.383 (2)	C42—H42	0.9300
C19—C20	1.384 (2)	N1—N2	1.3735 (18)
C20—C21	1.379 (2)	N5—N6	1.4047 (16)
C20—H20	0.9300	N6—H6N	0.8852
			
N2—C1—C31	121.91 (14)	C22—C23—C24	120.17 (17)
N2—C1—C2	113.08 (13)	C22—C23—H23	119.9
C31—C1—C2	124.98 (14)	C24—C23—H23	119.9
C1—C2—C25	112.28 (12)	C23—C24—C19	120.18 (16)
C1—C2—C3	99.59 (11)	C23—C24—H24	119.9
C25—C2—C3	113.38 (12)	C19—C24—H24	119.9
C1—C2—H2	112.3	C30—C25—C26	118.31 (15)
C25—C2—H2	109.5	C30—C25—C2	119.60 (15)
C3—C2—H2	109.5	C26—C25—C2	122.06 (14)
N1—C3—C4	112.51 (11)	C25—C26—C27	120.64 (16)
N1—C3—C2	100.88 (11)	C25—C26—H26	119.7
C4—C3—C2	109.70 (11)	C27—C26—H26	119.7
N1—C3—H3	110.5	C28—C27—C26	120.27 (18)
C4—C3—H3	110.6	C28—C27—H27	119.9
C2—C3—H3	112.3	C26—C27—H27	119.9
N3—C4—C11	120.59 (13)	C29—C28—C27	119.59 (16)
N3—C4—C3	118.25 (13)	C29—C28—H28	120.2
C11—C4—C3	121.03 (12)	C27—C28—H28	120.2
N3—C5—C10	121.45 (13)	C28—C29—C30	120.39 (18)
N3—C5—C6	119.09 (14)	C28—C29—H29	119.8
C10—C5—C6	119.45 (14)	C30—C29—H29	119.8
C7—C6—C5	119.86 (16)	C25—C30—C29	120.76 (18)
C7—C6—H6	120.1	C25—C30—H30	119.6
C5—C6—H6	120.1	C29—C30—H30	119.6
C6—C7—C8	120.60 (15)	C32—C31—C36	118.45 (17)
C6—C7—H7	119.7	C32—C31—C1	120.90 (17)
C8—C7—H7	119.7	C36—C31—C1	120.55 (15)
C9—C8—C7	121.02 (16)	C33—C32—C31	120.6 (2)
C9—C8—H8	119.5	C33—C32—H32	119.7
C7—C8—H8	119.5	C31—C32—H32	119.7
C8—C9—C10	119.49 (16)	C34—C33—C32	120.5 (2)
C8—C9—H9	120.3	C34—C33—H33	119.7
C10—C9—H9	120.3	C32—C33—H33	119.7
N4—C10—C5	120.74 (13)	C33—C34—C35	120.0 (2)
N4—C10—C9	119.73 (14)	C33—C34—H34	120.0
C5—C10—C9	119.50 (14)	C35—C34—H34	120.0
N4—C11—N5	119.19 (13)	C34—C35—C36	120.2 (2)
N4—C11—C4	123.01 (13)	C34—C35—H35	119.9
N5—C11—C4	117.69 (12)	C36—C35—H35	119.9
C13—C12—C17	119.61 (14)	C35—C36—C31	120.19 (19)
C13—C12—N5	121.14 (12)	C35—C36—H36	119.9
C17—C12—N5	119.24 (14)	C31—C36—H36	119.9
C12—C13—C14	119.81 (15)	C38—C37—C42	119.39 (17)
C12—C13—H13	120.1	C38—C37—N1	120.72 (16)
C14—C13—H13	120.1	C42—C37—N1	119.85 (18)
C15—C14—C13	120.61 (18)	C37—C38—C39	119.8 (2)
C15—C14—H14	119.7	C37—C38—H38	120.1
C13—C14—H14	119.7	C39—C38—H38	120.1
C14—C15—C16	119.61 (17)	C40—C39—C38	121.2 (3)
C14—C15—H15	120.2	C40—C39—H39	119.4
C16—C15—H15	120.2	C38—C39—H39	119.4
C15—C16—C17	120.62 (15)	C39—C40—C41	119.3 (2)
C15—C16—H16	119.7	C39—C40—H40	120.4
C17—C16—H16	119.7	C41—C40—H40	120.4
C16—C17—C12	119.71 (16)	C40—C41—C42	121.4 (3)
C16—C17—H17	120.1	C40—C41—H41	119.3
C12—C17—H17	120.1	C42—C41—H41	119.3
O3—C18—N6	122.41 (14)	C37—C42—C41	118.9 (2)
O3—C18—C19	122.20 (15)	C37—C42—H42	120.5
N6—C18—C19	115.39 (14)	C41—C42—H42	120.5
C24—C19—C20	119.13 (15)	N2—N1—C37	119.55 (13)
C24—C19—C18	122.71 (14)	N2—N1—C3	111.11 (12)
C20—C19—C18	118.14 (14)	C37—N1—C3	126.24 (14)
C21—C20—C19	120.29 (16)	C1—N2—N1	108.52 (12)
C21—C20—H20	119.9	C4—N3—C5	117.51 (13)
C19—C20—H20	119.9	C11—N4—C10	116.54 (13)
C22—C21—C20	120.18 (18)	N6—N5—C11	115.65 (11)
C22—C21—H21	119.9	N6—N5—C12	113.80 (11)
C20—C21—H21	119.9	C11—N5—C12	119.36 (12)
C21—C22—C23	120.03 (18)	C18—N6—N5	117.56 (12)
C21—C22—H22	120.0	C18—N6—H6N	120.2
C23—C22—H22	120.0	N5—N6—H6N	115.9

### Hydrogen-bond geometry (Å, º)

**Table:**

*D*—H···*A*	*D*—H	H···*A*	*D*···*A*	*D*—H···*A*
C2—H2···O3	0.98	2.28	3.2425 (19)	169
N6—H6*N*···N4^i^	0.89	2.30	3.1754 (18)	172
C16—H16···N2^ii^	0.93	2.56	3.4799 (19)	172

Symmetry codes: (i) −*x*, −*y*, −*z*+1; (ii) *x*−1/2, −*y*+1/2, *z*−1/2.
